# The impact of providing care for physical health in severe mental illness on informal carers: a qualitative study

**DOI:** 10.1186/s12888-024-05864-3

**Published:** 2024-06-06

**Authors:** Dolly Sud, Eleanor Bradley, Jonathan Tritter, Ian Maidment

**Affiliations:** 1 Aston University, Birmingham, United Kingdom; 2 Leicestershire Partnership NHS Trust, Leicester, United Kingdom; 3 University of Worcester, Worcester, United Kingdom; 4 Nord University, Bodø, Norway

**Keywords:** Severe mental illness, Schizophrenia, Bipolar, Parity of esteem, Metabolic

## Abstract

**Background:**

People with severe mental illness (SMI) such as schizophrenia and bipolar disorder are at a substantially higher risk of premature death in that they die between 10 and 20 years earlier than the general population. Cardiovascular disease (CVD) and diabetes are the main potentially avoidable contributors to early death. Research that explores the experiences of people with SMI highlights their struggles in engaging with health professionals and accessing effective and timely interventions for physical health conditions. A consequence of such struggles to navigate and access physical healthcare results in many people with SMI relying heavily on support provided by informal carers (e.g., family members, close friends). Despite this, the experiences of informal carers, and the roles they undertake in relation to supporting the physical health and psychotropic medication use of people with SMI, remains under-researched.

**Aims:**

To explore the impacts of providing care for physical health in severe mental illness on informal carers.

**Method:**

Thematic analysis of semi-structured interviews with eight informal carers of people with SMI in United Kingdom (UK) national health services.

**Results:**

Informal carers played an active part in the management of the patient’s conditions and shared their illness experience. Involvement of informal carers was both emotional and practical and informal carers’ own lives were affected in ways that were sometimes deeply profound. Informal carers were involved in both ‘looking after’ the patient from the perspective of doing practical tasks such as collecting dispensed medication from a community pharmacy (caring for) and managing feelings and emotions (caring about).

**Conclusions:**

Providing care for the physical health of someone with SMI can be understood as having two dimensions - ‘caring for’ and ‘caring about’. The findings suggest a bidirectional relationship between these two dimensions, and both have a cost for the informal carer. With appropriate support informal carers could be more actively involved at all stages of care without increasing their burden. This should be with an awareness that carers may minimise the information they share about their own needs and impacts of their role to spare the person they care and themselves any distress.

**Supplementary Information:**

The online version contains supplementary material available at 10.1186/s12888-024-05864-3.

## Introduction

People with severe mental illness (SMI) e.g., schizophrenia, bipolar disorder have higher morbidity and mortality than the general population [1] with their life expectancy reduced by between 10 and 20 years [2, 3]. Physical health conditions account for the overwhelming majority of premature deaths [4–6] and cardiovascular disease (CVD) [7], metabolic syndrome [8] and diabetes [9] are the main potentially avoidable contributors [4].

These physical health conditions can be attributed to a broad range of factors, including iatrogenic (adverse effects of medication e.g., weight gain) and factors related to the illness itself [10–12]. In addition, lifestyle related factors including reduced physical activity and increased rates of smoking [8]. Published research indicates that people with SMI receive a poor quality of care for their physical health. This includes low rates of regular [1, 2] comprehensive physical health checks [13, 14], gaps between mental and physical health service provision and between primary and secondary care [15] and a lack of involvement in physical health care discussions within mental health care planning [16].

Studies report that individuals with schizophrenia who have severe negative symptoms are less physically active and thus have lower aerobic fitness levels [17, 18]. In people with bipolar disorder, mood state has been reported as a barrier to physical activity [19]. People living with SMI are also more likely to experience financial deprivation; the multidimensional effects of this can influence on decision-making and judgement about lifestyle behaviours, including choices about exercise and nutrition [20].

Physical healthcare provision for people with SMI may also be influenced by attitudes and perceived clinical competencies of healthcare professionals. Studies report that general practitioners (GPs), community pharmacists and nurses working in primary care do not feel they have sufficient knowledge and skills to manage the physical health of people with SMI and desire more training [21–23]. One study [24] reported that the majority of 430 GPs interviewed did not or only partially believed that individuals with schizophrenia were reliable in referring their physical (82.3%) or mental health (77.1%) problems to doctors.

Research that explores the experiences of people with SMI highlights their struggles in engaging with mental health professionals and accessing effective and timely interventions for physical health conditions [22, 23]. Multiple studies report patients’ experiences of health professionals not taking their physical health concerns seriously, diagnostic overshadowing, disagreeable attitudes and a lack of relational skills [25–28]. A narrative review of international qualitative and quantitative studies including people with SMI and healthcare professionals concluded that stigma and the gaps and disconnect between mental and physical health services were the main barriers to accessing physical health care [29]. People with SMI also report a lack of confidence and ability to be assertive with their communication in acute and general medical health services which impacts negatively on their ability to negotiate for medical interventions [21].

A consequence of such struggles to navigate and access physical healthcare results in many people with SMI relying heavily on support provided by informal carers (e.g., family members, close friends) [30]. Despite this, the experiences of informal carers and the roles they undertake in relation to supporting the physical health and psychotropic medication use of people with SMI, remains under-researched [30, 31]. Clinical guidelines, policies and published research acknowledge the role of informal carers in supporting the mental health of people with SMI including increasing engagement with services [32–37]. The contribution made by informal carers to the support of family members with SMI in relation to their mental health is well recognised [36, 38] however, their role in supporting the physical health of their family members is often overlooked by healthcare providers and policy makers [31, 39]. Furthermore, recommendations for informal carers to be included in policy development initiatives for physical health in people with SMI is poorly realised, for example, lack of participation in care planning [40].

In the UK, National Institute of Health and Care Excellence (NICE) guidance [32] recognises that the informal carers of patients with SMI are important in assessment, engagement and in the long-term successful delivery of effective treatment. Furthermore, this guidance also highlights that informal carers have views and preferences that should be acknowledged alongside those of patients. The guidance from NICE [32] refers many times to the need for health services to provide support for informal carers as well as for patients, for example, it suggests providing informal carers with written and verbal information in an accessible format about diagnosis and management of the SMI. Thus, there is growing recognition that carers have needs that should be addressed. However, to date, the nature of the activities and needs of informal carers of patients with SMI with comorbid physical health illness and psychotropic medication and its adverse effects have been relatively little studied and understood.

It has long been acknowledged by health researchers that patients’ illness experiences cannot be appreciated as individual socially isolated phenomena [41, 42]. In other words, all illnesses are socially constructed at the experiential level, based on how individuals come to understand and live with their illness. Collectively, there is a paucity of published literature providing a comprehensive and contextualised understanding of the impact of the physical health conditions and psychotropic medication for SMI on informal carers’, spouses, partners, other family members and close friends [43].

The terms ‘carer’ and ‘informal carer’ are contested, definitions vary across policy and research. The criteria for defining an ‘informal carer’ in research are not established and are frequently culturally specific [44]. These terms are generally used to refer a person who provides unpaid, ongoing support with daily activities for someone living with a long-term condition or disability such as schizophrenia, outside of a formal, professional framework [45, 46] Informal carers are commonly family members but can also be neighbours and friends [45, 47, 48]. Furthermore, It is recognised that family carers may not self-identify as ‘(informal) carers’ [49].

## Method

In this research we gave primacy to participants’ own definitions of ‘(informal) carer’ and this was reflected in our approach to recruitment. Participants were either those who self-identified as someone who provided a significant amount of support for a person with SMI or were nominated as someone who provided a significant amount of support by someone with SMI. Throughout this paper the terms ‘informal carer’ and ‘carer’ for ease of reading are used to indicate these participants. In addition, the terms person with SMI and patient are used interchangeably, this is in keeping with how individuals in the research referred to themselves [23].

### Study design and eligibility

An exploratory qualitative study design that followed the Consolidated Criteria for Reporting Qualitative studies (COREQ) guidelines were employed [50] to structure the research design, analysis, and reporting of findings. Qualitative semi-structured interviews were conducted with eight informal carers. Individuals were eligible to take part if they were (i) ≥ 18 years and (ii) either self-identified as someone who provided a significant amount of support for a person with SMI or were nominated by someone with SMI as someone who provided a significant amount of support and (iii) the person with SMI had cardiometabolic risk, metabolic syndrome or a related disease e.g., diabetes.

With regards the semi-structured topic guide: (i) it was written considering which core event or series of events that might capture the phenomena of interest, (ii) questions were ordered to optimise an intuitive and conversational flow and (iii) it was refined through a series of reviews and piloting. There are various methods that are available to analyse qualitative data; thematic analysis allows for patterned meanings and common perspectives to be identified from respondents. Both of these together resulted in a holistic understanding of informal carer experience.

After enrolment each participant was asked to provide informed consent, reimbursed for out-of‐pocket and travel expenses and offered a £10 gift voucher as recognition for study participation. This study was a component of a larger study exploring patients’ lived experience of physical health conditions [23]. Ethical approval was granted by a National Health Service Research Ethics Committee (17/EE/3057).

### Sampling

Convenience sampling, purposive sampling, and snowball sampling were all used to recruit participants from across the United Kingdom using posters placed in clinics and patient areas within one mental health NHS trust, and more broadly on patient and public involvement websites, mental health websites and social media (Twitter®). This combination ensured we captured variation around the phenomenon under study and therefore maximised the potential contribution of inductive data analysis alongside a priori knowledge and understanding to our analysis and findings; the use of this approach allowed us to explore multiple viewpoints [51]. All participants provided written consent and were interviewed on the telephone or in person at a time and place of their choosing.

A provisional anticipated upper sample size that might potentially generate adequate data that would provide rich, complex and multifaceted data about patternings related to the phenomena of interest was estimated [23]. The lead researcher (DS) then made an in-situ decision about the final sample size, shaped by the adequacy, richness and complexity of the data collected for addressing the research question.

### Data collection

Between October 2018 and March 2020 semi-structured interviews were conducted by DS based on a flexible topic guide (Appendix One) designed to explore participants’ experiences of providing care for someone with SMI, the impact of psychotropic medication, adverse effects and physical health conditions. The topic guide was informed by a literature review [23, 52] clinical background and experience of the lead researcher and a pilot interview with an informal carer.

A non-judgmental, open questioning style was adopted, and participants were encouraged to introduce issues of importance not covered by the topic guide. Interviews were recorded using an encrypted digital recorder and transcribed verbatim by DS and a professional transcribing service. Identifying information was removed at transcription after which recordings were destroyed. Interviews averaged 45 minutes (range 30–60 minutes). Data generation and analysis occurred in parallel using a constant comparative technique with incoming data informing subsequent interviews [51].

The in-situ decision on the final sample size was shaped by the adequacy, richness and complexity of the data collected for addressing the research question. The decision was made within the process of data collection, reviewing data quality during the process and data saturation [53, 54].

### Data analysis

Data were analysed on paper using thematic analysis (TA) exploring participants’ lived experiences, perspectives, behaviour and practice; the factors and social processes that influenced and shaped particular phenomena [55] were central to the research. Throughout the process of analysis the need to maintain quality and relationship between analytic practices was prioritized. Furthermore, attention was paid to the research framework and research question being answered, as well as the use of TA with clear purpose and intention and in a sensitive and reflexive way that respected the data collected [23, 56].

Initial familiarisation with the data was achieved by reading each transcript several times followed by line-by-line coding and then clustering codes to form themes. DS read all the transcripts with input from the research team who met regularly throughout to review and discuss the analysis. The team comprised academic researchers who have extensive experience of research across areas of psychiatry. In addition, two of the authors also have extensive experience as healthcare professionals in psychiatry.

## Results

Eight informal carers participated in the interviews. All informal carers were white (Table 1).

**Table 1:** Demographic data of informal carers of people with severe mental illness and physical health conditions (self-reported)

Participant pseudonym	Gender	Age range	Relationship to the patient they care for	Physical health conditions of the patient they care for	Psychiatric diagnosis/es of the patient they care for	Do they reside with the patient they provide care for?
Daniel	Male	40–44	Was the patient’s fiancé/ romantic partner	Smoker	Bipolar Disorder	Did live together at the time of the romantic relationship
Louisa	Female	65–69	Sister	Pre-diabetes Obese	Schizophrenia	No
Scott	Male	55–59	Husband	Overweight	Schizoaffective Disorder	Yes
Naomi	Female	50–54	Mother	Morbidly Obese Ex-smoker	Schizophrenia, Obsessive Compulsive Disorder Autism Attention Deficit Hyperactivity Disorder	Yes
Charles	Male	65–69	Romantic partner	Overweight	Paranoid Schizophrenia Obsessive Compulsive Disorder	No
Mikkel	Male	40–44	Brother	Obese	Schizophrenia	No
James	Male	40–44	Husband	Overweight	Bipolar Disorder Post-Traumatic Stress Disorder	Yes
Julia	Female	25–29	Daughter	Hypertension Obese	Antibiotic Induced Psychosis	No

**Fig. 1:**
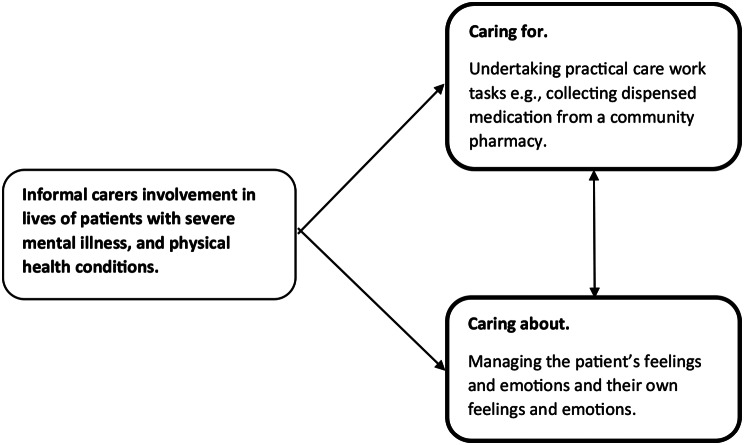
Themes identified from the analysis of qualitative data collected from eight informal carers of patients with SMI

Informal carers in the study played an active part in the management of the patient’s conditions and shared their illness experience. Informal carers’ involvement was both emotional and practical and informal carers’ own lives were affected by the patient’s journey in ways that were sometimes deeply profound. Informal carers were involved in both ‘looking after’ the patient from the perspective of doing practical tasks such as collecting dispensed medication from a community pharmacy (caring for) and managing feelings and emotions (caring about) (Fig. 1).

There were times in the patient journey when more practical informal care work was required, for example, in supporting patients attending appointments. In contrast to the more continuous need for emotional support and being ‘cared about’ that constituted a significant proportion of informal care work. Informal carers in this research had little choice but to engage in often arduous emotion work both to help the patient and to manage their own worries, anxiety or distress.

*I feel a sense of responsibility but more than like I have no choice…I feel obliged to keep morale high and manage the negative effects on feelings. If I run away from it then this effect would be worse because I would be making it into much more of a problem than it is. As long as I keep cheerful and happy then their morale is much higher….no point in sitting and dwelling in the worry or anxiety that comes with being a carer. On the other hand I think I would find it more difficult if I don’t do things to cope with it myself. If I leave my emotions unmanaged then they would get me down and that would impact on the both of us* (James).

### Caring for: undertaking practical care work tasks

Diagnosis of SMI and/or starting psychotropic medication led to changes in the type and allocation of practical care work tasks within the household. Informal carers found themselves involved in a variety of new caring work. These changes tended to happen immediately following diagnosis and/or starting psychotropic medication.

*Within a matter of months everything at home just changed. It was overwhelming at first. I was doing all the shopping, cleaning, cooking and washing as well as the ironing. I was exhausted! I just remember that she just wasn’t able to do any of that stuff at that time- we were grappling with trying to get everything stable and settled for her – the diagnosis and taking the medication at the right time every day. I had no time to think about anything else.* (Scott).

The quantity and nature of care work changes over time as levels of impairment due to SMI or physical health conditions or adverse effects from the psychotropic medication altered. Examples given by respondents included activities of daily living such as personal hygiene. Higher levels of practical care work were required when there was a mental health relapse, or the burden of adverse effects or physical health problems increased. Here the emotional impact of practical care work can also be seen.

*He’s been so ill recently, so incredibly unwell but he managed to have a shower to have a wash. When he came downstairs. I could see that his toenails were incredibly long. I said to him, ‘You need to cut your toenails.’ and he said, “I can’t reach them’.”* Naomi went on *“That is desperately sad, isn’t it? That at 26, you can’t, physically, cut your own toenails, that your mum’s got to cut your toenails because you can’t actually reach them because you’ve gained so much weight.* (Naomi).

For some, practical care work was already a significant feature of their lives before the patient’s diagnosis of SMI and the subsequent commencement of psychotropic medication because they had other caring responsibilities such as having children in their household or provided care for others in their family or social networks. The diagnosis of SMI and managing the medication made things worse.

*When you’ve got two young children and you’re on a low income, it’s just…it’s a huge amount of work just getting by, paying the bill, making sure the housework is done, meals are cooked…… it takes its toll and when you’ve got the additional problems on top of that, my son’s issues and (patient name)’s issues and all the health problems, well, it’s even harder.* (Scott).

Practical caring responsibilities for other relatives in the household added to informal carers’ difficulties and fatigue:

*Well, I’ve got two small kids aged 10 and 4. They have their own needs. I find that I sometimes have to spread myself quite thinly to manage everything, looking after my sister especially when things are tough for her, it’s a lot to coordinate and I sometimes feel I have nothing left in the tank.* (Mikkel).

Adding to the complexity of the issue, some informal carers had long term conditions of their own that required care. The needs of informal carers of patients with SMI can often be submerged by the need to care:

*Well, on top of everything else to do with (patient) I’ve got my own health problems to deal with, I was diagnosed with diabetes…there are appointments at the GP surgery, with the podiatrist and the ophthalmologist and a bunch of tablets to take. I’ve been told that I need to be careful with my diet and I need to lose weight. I know that my own mood has been low as a result.* (James).

The patient receiving a diagnosis of an SMI, comorbid physical health illness or experiencing significant adverse effects of psychotropic medication had implications not only for the responsibilities of informal carers but also a denial of practical support that they might have otherwise received from the patient. At these times the informal carer, therefore, may in turn require others to care for them. Like a chain reaction, having a knock on effect on practical care work tasks can disrupt the lives of many others.

*Now, I can’t just expect or ask her (patient) to help me with things the way she used to before, before the diagnosis and before things got bad for her with her health. My friends and people in my family go very far above and beyond to give me the help I need compared to before. So like some of the practical stuff like if I need a lift to see the diabetes specialist at the hospital which is far away from where I live. It’s been quite a bit of change.* (James).

Informal carers of people with SMI are therefore often embedded in sets of caring relationships requiring both giving and taking. Furthermore this illustrates that there is reciprocity between ‘patients’ and ‘carers’ in caring relationships.

Household tasks such as cleaning, shopping and cooking were most frequently noted. Informal carers often commented on their consequential tiredness:

*Every day there was like a hundred things to do, tidying up, getting to the shops to get food and then making sure everyone had eaten. At one time we could rely on (patient) to do those things. I felt pretty much tired to exhausted all the time. But I suppose our life had changed, it was like a matter of weeks after (patient) got diagnosed and started medication, it has just totally changed, I had to do more and more and things around the house things just got harder and harder.* (Scott).

What can also be seen very clearly is that the practical and emotional aspects of caring are linked. We shall now turn our attention to provision of emotional support.

### Caring about: management of feelings

The management of feelings and emotions is a critical aspect of what informal carers do in the context of SMI, comorbid physical health and psychotropic medication and their adverse effects. Although the distinction has been made between informal carers managing the feelings of the patient and managing their own feelings they are inextricably connected.

One aspect of this emotion work was trying to make sure that the patient did not feel alone or neglected. This centred on ‘being there’ for the patient, and in many cases providing reassurance that they would ‘face things together’. Keeping positive and ‘things being normal’, or as normal as possible, also featured in emotion work.

*Well, for us carers a lot of it’s about trying to keep things as normal as possible for them. For me, I found I had to toughen up, to be there and be consistently consistent with a positive outlook no matter what is happening to keep things in balance.* (Daniel).

Informal carers said they felt hope, trust and confidence in both the future and the healthcare being provided as expressed by the following carer:

*You’ve got to think of it like this, these psychiatrists are experts, they manage all this, the medication, the side effects, the psychotic relapses like someone eats breakfast every day. It’s an everyday thing to them. I’ve got to try and be the same, just carry it, look forward, show that I am optimistic about the future. It helps (patient) think in a positive way too if I do that* (Daniel).

Concurrent to managing the emotions of patients, informal carers also had to manage their own feelings and emotions: doing emotion work on themselves. An analytical distinction was made between these two categories but they are fundamentally interlinked: managing the feelings of the patient so that they felt better and vice versa. Informal carers spoke about this in terms of having to ‘be strong’ and to avoid ‘giving in’ toemotion.

Some carers expressed that there were expectations about how they should behave. This suggests an awareness of ‘normative guidelines’ or ‘social feeling rules’ in relation to the ‘proper thing to do’ under the circumstances, for example, putting the patient first which created an additional and significant emotional burden for the carer:

*And the community psychiatric nurse asked me “just how are you coping?”. I said, “I don’t know”. They said, “well you’ve gotta be strong”, they said “you’ve just gotta be strong”. And I thought afterwards, when (patient) came home, I thought, well, why have I got to be strong? Why have I got to be strong, because I’m as vulnerable as anybody else? And I feel like I’m taking it all on my shoulders.* (Julia).

Carers often struggled to live up to these expectations inevitably increasing the burden on them. Failing to live up to this idealised expectation of the “perfect” carer created feelings of guilt. Informal carers also expressed worries about the future. Concerns that it was not possible to always share with the patient.

*When you’re tired you’re less able to deal with sometimes the irrationality of a lot of stuff that you’re having to deal with and watch all the stuff that your loved one is having to deal with and all the stuff you’re both having to deal with together. I’m not superhuman…. I am dealing with a range of emotions on a daily basis, and you know, from being incredibly frustrated and holding it all in on a good day, most days to on occasion saying to him what is he going to do when I am not around, asking him how he’s going to cope when I am not here…I feel so guilty for doing this…* (Naomi).

Some informal carers said that they felt unable to express their own need for emotional help or support. This included seeking help from spiritual or religious advisors, healthcare professionals or family or social networks. However, expressing their own needs was difficult when the practical or emotional needs of the patient were high.

*I dunno, when things get bad for him I just feel like my needs and my feelings aren’t that important or significant in the grand scheme of things. I feel like it would be selfish of me to ask for help for myself at those times when he’s going through such a rough time of it. Anyway, even if I did there isn’t the space to manage both of our needs when that happens. My needs pale into insignificance in comparison.* (Naomi).

## Discussion

The findings of this study highlight that additional care work demands were a significant and important feature of informal carers’ lives in the context of SMI, although this varied with the stage of the patient’s diagnosis, starting medication, and the presence of co-morbidity in patients. Informal carers’ own morbidity status, alongside their social and material circumstances, had a significant bearing on their ability and capacity to take on greater amounts and types of often complex care work. Informal carers expressed a particular need for help with everyday practical tasks and the personal effects of their care burden.

Findings here also suggest that there is reciprocity and co-dependency between ‘patients’ and ‘informal carers’ in caring relationships. Furthermore that there may be greater patient dependency during periods, for example, after starting medication. The concept of reciprocity between informal carers and patients with SMI is explored in our previously published paper [57].

Literature on the impact of significant health conditions reports that these can disturb established divisions of labour within and beyond households [41, 42]. Alteration and variability in the quantity and quality of care work in relation to severity of illness or degree of impairment found in this research is also reflected in the broader literature. Informal carers’ own morbidity along with their social circumstances had an impact on their capacity to take on greater amounts of care work.

Half of respondents in this study were partners of individuals with SMI. This may be important in interpretation of the findings. For example, it is known that partner carers frequently experience difficulties and confusion in discerning between romantic roles and caregiving roles. This may have implications in their experiences of physical health conditions in the person they provide care for [58]. Studies which recruit a larger number of participants are needed to explore this phenomenon in more detail.

The management of feelings and emotions is a critical aspect of what informal carers do in the context of SMI, comorbid physical health and psychotropic medication and their adverse effects. This constitutes ‘emotion work’ [59]; the emotional effort made by an individual to manage their own feelings and those of others. Doing emotion work in the context of SMI, comorbid physical health and psychotropic medication appears to be fundamentally about the management of feelings in order to facilitate a sense of control over events, which are frequently outside of their control.

Informal carers were faced with supporting the emotional needs of their family member (the patient), whilst also finding ways to cope with their own emotions. Unsurprisingly, these aspects of ‘caring about’ themselves and others were inextricably linked. There was a feeling amongst some carers in this study of an obligation to be ‘strong’ and ‘positive’ and to maintain a sense of ‘things in life to continue on as normal’.

This need to be strong, to cope, and to maintain a sense of normality for the family created a significant burden on the carer; a similar pattern as found among informal carers for people with Alzheimer’s disease and older people in general [60, 61]. In part, this emotion work distinguished between the two different but connected aspects of caring work: caring for and caring about. Emotion work was an aspect of both types of caring work but was the dominant feature of the work of caring about.

As part of the caring process informal carers can be seen as sharing the illness and the difficulties this entails becomes a joint experience. Grounded in their knowledge of the patient’s disposition, informal carers sometimes glossed over or withheld information, for example, about their own emotional and practical needs or the impact of changes in amount of practical work they were doing in order to minimise distress for the patient and themselves.

Our findings relating to emotion work undertaken by informal carers reveals that they may often prioritise the interests and needs of patients before their own. This can result in burnout and additional morbidity as those who provide the care end up needing interventions and care themselves. This at the very least creates a tension or at worst a double bind; being an informal carer creates health needs for the carer themselves. Caring has a cost, both emotional and physical.

These research findings have implications for those working with people living with SMI and their families, including those involved with treatment decisions. They suggest that providing support to informal carers is as important as that for the patient themselves. Conceptualising the informal carer as an active agent co-constructing the illness experience of the patient can provide insights into the roles played by and the needs of informal carers. Healthcare professionals could work more actively with carers to further embed their experiences in care decisions, given that the consequences of these decisions often fall to them [62].

Informal carers may be less likely to accept support services directed at them. They may feel that a consequence of doing would result in attention and resources being redirected away from the patient they are providing care for. This is corroborated by findings of other research [63, 64]. Healthcare providers may have to reassure informal carers that they have legitimate and justifiable needs and that their contribution to care for the patient is substantiable and critical: in short that their needs count.

The findings here are concordant with those of previous qualitative research which report that informal carers have essential roles in undertaking practical care work tasks to support physical health of people with SMI [43]. However, in this study we report on the management of emotions and caring for which was absent from those studies. Previous studies have highlighted that informal carers are not fully involved in patients’ physical health care and their contributions to optimize physical healthcare outcomes do not appear to be completely recognised [43, 65]. Our study adds that in doing so it is important to consider how informal carers may minimise the impact of providing care on themselves. Consideration of these issues are important with regards updates to clinical practice and policy in this area.

### Strengths

The background and discipline of the lead and co-authors is relevant; they have the appropriate methodological and clinical expertise and are experts in the field that is the focus of the research presented here. The lead author is a clinical academic pharmacist; their PhD explored the lived experience of physical health in severe mental illness and their 25 years of clinical practice has been split almost equally between general medicine and psychiatry. The supervisory team is multidisciplinary (pharmacy, health psychology, sociology, and public policy) and have extensive experience of conducting applied research within mental health settings.

Recruiting nationally from across the UK is another strength as respondents will be from more geographically diverse areas and therefore have diverse experience of health and social care.

In recent years, a greater emphasis has been placed on experiences in and of healthcare. As such, qualitative research such as this, which seeks to understand informal carers’ perspectives of a group, has recognition as being vitally important in health services research. The merit of qualitative research lies in its utility to capture information about beliefs, values and attitudes that drive behaviours by drawing out answers to questions that ask what, how and why [66]. Using the information gathered about experiences provided by individual members of a group from different contexts, in this case informal carers, this research like other qualitative research relies on deductive and inductive reasoning to generate broad themes or general rules from which observations about a phenomenon are generated [66].

Analytical rigour is required to prevent bias and ensure that findings reflect the data [23]. There are challenges in analysing narratives from semi-structured interviews because of the incongruence between the practical process of data analysis while ensuring quality and maintaining both ethical or theoretical standpoints [23]. Delineating the boundaries of a story in relation to interview data is often challenging in qualitative research. The narratives that were analysed in this research were co-constructed with participants. The way data is analysed, and findings are produced depends on the particular perspective of the researcher and disciplinary framing.

The purpose of this research was to document and analyse research participants’ views, experiences, and perceptions. Therefore, analysis required a repeated process of immersing and re-immersing in the co-constructed narratives to ensure that participants’ stories were dominant, and the respondent experience remained central to the research. This involved reading and re-reading the transcripts, extended engagement with the narrative content to identify themes and subjecting the results of the analysis to critical review by all authors. Respondent triangulation helped to capture different viewpoints and understandings of the phenomenon under investigation, resulting in a more complete picture as well as enhancing the validity of the collected data [23].

### Limitations

This research was conducted with a small number of informal carers from the UK. There is also an implicit limitation in how the study was promoted and recruitment was conducted, people who respond may generally have strong opinions about the issue in question. Individuals’ views were influenced by experiences of specific health services in the UK. It is important to note that all the informal carers in this study were from white ethnicity and the findings may not be applicable to individuals from other ethnic groups. Further research with a larger sample of carers from a range of ethnicities and geographical settings should be prioritised.

The research presented here, however, can offer an initial understanding of some of the issues experienced by informal carers and serve to undertaking further focused and hypothesis-driven investigation.

The findings suggest that informal carers could be more actively involved at all stages of care, research is needed into how this might be done without increasing their burden, for example, what specific support would informal carers need and how best this could be provided. This would have to address issues around patient confidentiality and consent. Previous research has highlighted that informal carers acknowledge that patients have a right to confidentiality [67]. This research also highlights that some informal carers felt that even if the patient did not consent to their involvement, they should remain informed about the service user’s care and be involved in a way that did not breach confidentiality [67].

This study provides a preliminary understanding of the experiences of informal carers which can be further built upon with a larger, more representative sample size. Use of mixed methods research would further add to the knowledge base. Future research into the major challenges and barriers to management of physical health in people with SMI would provide transferable information to support patients and carers. Inclusion of measures of well-being and quality of life would further inform this subject.

According to the Centre for Reviews and Dissemination [68]: *“The relevance of qualitative research to the assessment of health interventions, especially those that are complex, has been recognised. As a result, qualitative and quantitative methods are increasingly being used together in primary evaluative research. The main reason for the adoption of mixed methods in primary research is to enhance relevance in the decision-making process.”* Such studies could use the findings of qualitative research already conducted to inform their study protocols.

The findings of this study are limited to the experience of cardiometabolic risk, metabolic syndrome and related diseases e.g., diabetes in SMI [69]. Future studies should focus on other long term health conditions e.g., cancer, airways disease that are known to be prevalent in SMI. In addition, research that focuses on preventive healthcare is needed.

The implications that patient physical health problems present for carer well-being and the quality of the caregiving relationship in psychosis deserve further investigation.

## Conclusion

Caring for the physical health of someone with SMI can be understood as having two dimensions - ‘caring for’ and ‘caring about’. The findings suggest a bidirectional relationship between these two dimensions, and both have a cost for the informal carer. With appropriate support informal carers could be more actively involved at all stages of care without increasing their burden. This should be with an awareness that carers may minimise the information they share about their own needs and impacts of their role to spare the person they care and themselves any distress. Finally, the findings touch a richer seam that needs attention, which is the personal cost or toll on the carer of managing their loved one’s illness.

### Electronic supplementary material

Below is the link to the electronic supplementary material.

Supplementary Material 1

## Data Availability

The datasets generated and/or analysed during the current study are not publicly available due to ethical issues involving participant’s data and privacy.
