# Supplementary material for: The impact of providing care for physical health in severe mental illness on informal carers: a qualitative study
Source: BMC Psychiatry. 2024 Jun 6;24:426. doi: 10.1186/s12888-024-05864-3 (PMC11154995; doi:10.1186/s12888-024-05864-3)
Supplement: Supplementary file 1 — Supplementary Material 1 [file 12888_2024_5864_MOESM1_ESM.docx]

Additional file 1

**CARDIOPHITNESS Informal carer interview topic guide. IRAS project ID 233121**

**Topic Guide for interview with Informal carer_v 1.3 30/12/2018**

**Topic Guide for interview with informal carers – ORIGINAL**

1. **Introduction to interview**

- Thank you for participating
- Introduction of lead researcher and others involved in the research (Aston University and LPT NHS Trust)
- Overview of research and purpose.
- Approximate duration of the interview is 45-60 minutes
- Proposed structure of interview - interested in what is important to them; no right or wrong answers, it is their perspective that we are interested in
- Consent to participate and approval to audio record the interview
- Confidentiality, anonymity of interview data and right to withdraw at any time
- Collection of basic demographic data

1. **Questions**

- Introduction [Note: The aim here is to get an idea of the person behind the story. Focus on the “now” – their current life. Ask prompting questions to suit the person.]
- Could you tell me a little about yourself – Prompts: – What do you do (as in work, keeping themselves occupied etc.)? – What are your interests and hobbies? – Family and social networks?
- Considering the person you care for and the support you give them:

*(Idea of both physical health/wellbeing and mental health within this)*

- How would you describe the physical health/wellbeing of the person you care for at the moment? Have you got any concerns about their physical health/wellbeing?
- Tell me about the care they are getting for their physical health issue (used named example)**.**
- What things help the physical health of the person you care for? What things don’t help?
- Role of the pharmacist or pharmacy

*(explore both community and hospital pharmacy/pharmacist in relation to the person they care for)*

- How often to do you visit a pharmacy or speak to a member of the pharmacy team?
- What are the main reasons for visiting the pharmacy/speaking to the pharmacist?
- Does the pharmacy/pharmacist help you with the medicines of the person you care for?
- Does the pharmacy/pharmacist support their physical health/wellbeing? Are any of these for health promotion or risk reduction (name example as appropriate e.g., diet, smoking cessation)?
- Tell me about a time when the pharmacist has helped or given you advice about the physical health/wellbeing of the person you care for
- What help or support would you like the pharmacist/pharmacy to provide?
- What kind of relationship would you say you have with your pharmacist/pharmacy team?
- Potential barriers
- What things get in the way of the person you care for developing/improving their physical health/wellbeing?
- How do you think these might be overcome?
- What do you think might get in the way of pharmacy/pharmacists supporting the physical health of the person you care for?
- How do you think these might be overcome?
- Facilitators/enablers
- What things help develop/improve the physical health/wellbeing of the person you care for?
- How do they help?
- What would you like to make it easier for the person you care for to do things for their physical health/wellbeing?
- What would make it easier for the person you care for get support from the pharmacy/pharmacist?
- What would make it easier for pharmacies/pharmacist give the person you care for support for their physical health?
- Conclusions
- Are there any other things you would like to add to these discussions?
- Thanks for taking part in this research study and for your time.

____________________________________

Please note that this document is for the Lead Researcher their research team only and will not be given to participants.

**CARDIOPHITNESS Informal carer interview topic guide. IRAS project ID 233121**

**Topic Guide for interview with Informal carer_v 1.3 30/12/2018**

**Topic Guide for interview with informal carers - INCLUDES QUESTIONS AND COMMENTS THAT WERE ADDED DURING THE PROCESS OF GATHERING DATA AS PART OF THE ITERATIVE PROCESS**

1. **Introduction to interview**

- Thank you for participating
- Introduction of lead researcher and others involved in the research (Aston University and LPT NHS Trust)
- Overview of research and purpose.
- Approximate duration of the interview is 45-60 minutes
- Proposed structure of interview - interested in what is important to them; no right or wrong answers, it is their perspective that we are interested in
- Consent to participate and approval to audio record the interview
- Confidentiality, anonymity of interview data and right to withdraw at any time
- Collection of basic demographic data

1. **Questions**

- Introduction [Note: The aim here is to get an idea of the person behind the story. Focus on the “now” – their current life. Ask prompting questions to suit the person.]
- Could you tell me a little about yourself – Prompts: – What do you do (as in work, keeping themselves occupied etc.)? – What are your interests and hobbies? – Family and social networks **and groups**?
- Considering the person you care for and the support you give them:

*(Idea of both physical health/wellbeing and mental health within this)*

- How would you describe the physical health/wellbeing of the person you care for at the moment? **And emotional well-being?**
- **what do you see as the link between physical health and mental health? What does quality of life mean to you? In what way do you think it is important to the person you provide care for ?**
- Have you got any concerns about their physical health/wellbeing?
- Tell me about the care they are getting for their physical health issue (used named example) **Quality and type of support if relevant and support with medication etc.**
- What things help the physical health of the person you care for? What things don’t help?
- **What has the impact of mental health medication or a change in mental health medication change been on the physical health, your quality of life, health, or well-being of the person you care for?**
- **What information have you/they received about screening and management of your physical health in relation to your mental health medication? Are you aware of any regular physical health checks/appointments needed/attended?**
- **How do you manage any physical health side-effects such as weight gain or diabetes (as relevant) for the person you care for? How does it impact your life/everyday life?**
- Role of the pharmacist or pharmacy

*(explore both community and hospital pharmacy/pharmacist in relation to the person they care for)*

- How often to do you visit a pharmacy or speak to a member of the pharmacy team?
- Do you know how often does the person you care for visits a/their pharmacy or speak to a member of the pharmacy team?
- What are the main reasons for visiting the pharmacy/speaking to the pharmacist?
- Does the pharmacy/pharmacist help you with the medicines of the person you care for?
- Does the pharmacy/pharmacist support their physical health/wellbeing? Are any of these for health promotion or risk reduction (name example as appropriate e.g., diet, smoking cessation)?
- Tell me about a time when the pharmacist has helped or given you advice about the physical health/wellbeing of the person you care for
- What help or support would you like the pharmacist/pharmacy to provide?
- What kind of relationship would you say you have with your pharmacist/pharmacy team?
- **If they say they don’t go to the pharmacy at all ask – would you consider going to the pharmacy to ask for information or advice about medication the person you care for is taking/psychotropic medication?**
- Potential barriers
- What things get in the way of the person you care for developing/improving their physical health/wellbeing?
- How do you think these might be overcome?
- What do you think might get in the way of pharmacy/pharmacists supporting the physical health of the person you care for?
- How do you think these might be overcome?
- Facilitators/enablers
- What things help develop/improve the physical health/wellbeing of the person you care for?
- How do they help?
- What would you like to make it easier for the person you care for to do things for their physical health/wellbeing?
- What would make it easier for the person you care for get support from the pharmacy/pharmacist?
- What would make it easier for pharmacies/pharmacist give the person you care for support for their physical health?
- Conclusions
- Are there any other things you would like to add to these discussions?
- Thanks for taking part in this research study and for your time.

____________________________________

Please note that this document is for the Lead Researcher their research team only and will not be given to participants.
